# Menadione Contribution to the In Vitro Radical Scavenging Potential of Phytochemicals Naringenin and Lignin

**DOI:** 10.3390/ijms242216268

**Published:** 2023-11-13

**Authors:** Zvezdelina Yaneva, Donika Ivanova, Monika Toneva, Milena Tzanova, Vanya Marutsova, Neli Grozeva

**Affiliations:** 1 Department of Pharmacology, Animal Physiology, Biochemistry and Chemistry, Faculty of Veterinary Medicine, Trakia University, Students Campus, 6000 Stara Zagora, Bulgaria; donika.ivanova@trakia-uni.bg (D.I.); monika.toneva@trakia-uni.bg (M.T.); 2 Department of Biological Sciences, Faculty of Agriculture, Trakia University, Students Campus, 6000 Stara Zagora, Bulgaria; milena.tsanova@trakia-uni.bg (M.T.); n.grozeva@trakia-uni.bg (N.G.); 3 Department of Internal Diseases, Faculty of Veterinary Medicine, Trakia University, Student Campus, 6000 Stara Zagora, Bulgaria; vanya.marutsova@trakia-uni.bg

**Keywords:** vitamin K_3_, phytochemical, alkali lignin, naringenin, DPPH, ABTS

## Abstract

Vitamin K_3_ (menadione), classified as a pro-vitamin, is a synthetic form of the fat-soluble family of vitamin K compounds. The combination of the vitamin with other molecules sharing structural and/or functional similarities, such as naturally occurring polyphenols, vitamins, or biopolymers, could potentiate mutual improvement of their antioxidant activity. The aim of the present study was to evaluate the role and contribution of vitamin K_3_ to the in vitro radical scavenging capacity of double and triple combinations with the phytochemicals naringenin and lignin, as well as assess possible intermolecular interactions between the bioactive compounds. Comparative analyses of the DPPH and ABTS radical scavenging activity of the pure substances vitamin K_3_, naringenin, and lignin; the two-component systems lignin/vitamin K_3_ and vitamin K_3_/naringenin; and the triple combination vitamin K_3_/flavonoid/lignin were carried out. The experimental results demonstrated increased DPPH and ABTS activities of the vitamin in combination with lignin compared to those of the two pure substances, i.e., a synergistic effect was observed. The registered significant increases in the radical scavenging activity of the triple combination determined via both methods are indicative of a remarkable potentiation effect, i.e., higher antioxidant potential exceeding the additive activity of the three pure substances.

## 1. Introduction

Vitamin K_3_ (menadione, 2-methyl-1,4-naphthoquinone), classified as a pro-vitamin, is a synthetic form belonging to the vitamin K family that does not occur naturally, unlike the other two fat-soluble forms of vitamin K, namely vitamin K_1_ (phylloquinone) and vitamin K_2_ (menaquinone) [1,2]. Menadione may undergo one electron reduction, resulting in the formation of unstable free radicals, which, via a rapid reaction with oxygen, produce ROS, thus causing oxidative stress [3]. The action of menadione in the organism is not restricted to its applicability as a biosynthetic precursor to vitamins K_1_ and K_2_. According to the literature data, it exhibited various biological activities, such as antichagasic, anticancer [1,2,4], antifungal, antibacterial [5], antimalarial [6], and antiparasitic [7] potential [1,8], and it is classified as an essential nutrient involved in blood clotting and bone health [9]. The pro-vitamin was proven to be an effective inhibitor of lipid peroxidation in microsomes suppressing lipid peroxide formation via various mechanisms, including a relationship with single-electron transfer enzymes [10]. According to other studies, the reduced forms of vitamin K_3_ could display antioxidant activities [3,11,12,13].

Moreover, menadione potentiated aminoglycosides against multi-resistant bacterial strains of Staphylococcus aureus, Pseudomonas aeruginosa, and Escherichia coli, as it decreased the antibiotics’ minimal inhibitory concentration [14]. The applicability of the pro-vitamin as an additive in animal feedstock was also studied. A team of scientists proved that the dietary addition of adequate doses of vitamins A and K_3_ (7000 IU/kg and 2.0 mg/kg, respectively) improved the immune function and intestine antioxidant capacity of aged laying hens, and excessive levels did not cause superior effects [15].

Biomedical science fields need not only in-depth research into how vitamin K_3_ is involved in metabolic pathways but also evidence of its interaction with other bioactive substances and the arising resultant physiological/pharmacological effects [16]. In this respect, novel studies demonstrated that combining vitamin K_3_ with other structurally similar molecules found in polyphenol-rich Juglans regia [17] or vitamins or drugs that also function through the modulation of intracellular redox states could potentiate the overall antitumor effects of the combinations [18]. The studies of Lamson et al. (2010) and Bonilla-Porras et al. (2011) reported that a combination of the natural antioxidant vitamin C with menadione can enhance the cell-killing effect of an oxidizing anticancer system in vitro [19,20]. Consequently, performing systematic reviews and meta-analyses to gain a greater sense of understanding and clearer picture of vitamin K_3_ responses to human health is essential for modern scientific research [21].

Nowadays, there is growing interest in natural polyphenols of plant origin, such as flavonoids and biopolymers, due to their significant physiological activities [22,23]. Although the mechanisms through which flavonoids and bioheteropolymers act as antioxidant agents are not completely revealed, the roles of hydroxyl groups are accepted as being vital for their free radical scavenging potential. However, depending on the physiological and environmental conditions, the biological activity may be controlled by H acidity, phenoxide anions’ proton activity, ionization potential, intramolecular H bonds, phenolic O—H bond dissociation enthalpy, intramolecular H bonds, etc. [24].

Naringenin is a naturally occurring flavanone widely distributed in edible fruits like citrus species, tomatoes, bergamot, and figs [25]. It offers various pharmacological benefits, such as protective effects against cytochrome P450 3A4 activity, lipid peroxidation, lipoxygenases, and cyclo-oxygenases [24]. Prior investigations reported the ability of naringenin to safeguard cells after damage initiated by oxidative stress and inflammatory responses [26]. Moreover, antioxidant, antitumor, antiviral, antibacterial, anti-inflammatory, antiadipogenic, and cardioprotective effects have been ascribed to this phytochemical [27,28,29,30,31].

The natural heteropolymer lignin and its derivatives are acknowledged as alternative candidates for the design of novel medicinal products and drug-delivery formulations due to their valuable biological activities, such as antioxidant, anti-inflammatory, antidiabetic, antiviral, and antitumor effects [32]. In this context, antioxidant potential acquired as an inherent activity to polyphenolic compounds is accepted as the leading mode of action of lignin-like compounds [33].

While there are many reports of the biological activities of various natural agents and vitamins, there is lack of information on the biological and physiological behavior and effects of their combinations. A number of such compounds are classified as antioxidants and there has been both longstanding concern and expectation regarding how mixtures of these substances would affect the overall radical scavenging capacity of the complex systems [34,35].

Moreover, the total antioxidant potential of multicomponent systems cannot be forecast based on the antioxidant activity of their individual components; thus, the overall activity is usually explained through the existence of combined synergistic, antagonistic, or additive effects. Polyphenolic compounds are among the most powerful and widely studied antioxidants; however, information about their molecular interactions with other biologically active compounds, such as vitamins and biopolymers, is lacking.

In the current research, the experimental systems were limited to two and three core structures to excavate in detail their interactions in the context of molecular structure, redox reactivity, and redox-related bioactivities. The latter could enable better understanding and prediction of the radical scavenging potential of combinations between phytochemicals and a pro-vitamin [34]. The aim of the present study was to evaluate the role and contribution of vitamin K_3_ to the in vitro radical scavenging capacity of double and triple combinations with the phytochemicals naringenin and lignin, as well as to assess possible intermolecular interactions between the bioactive compounds.

## 2. Results and Discussion

**Figure 1:**
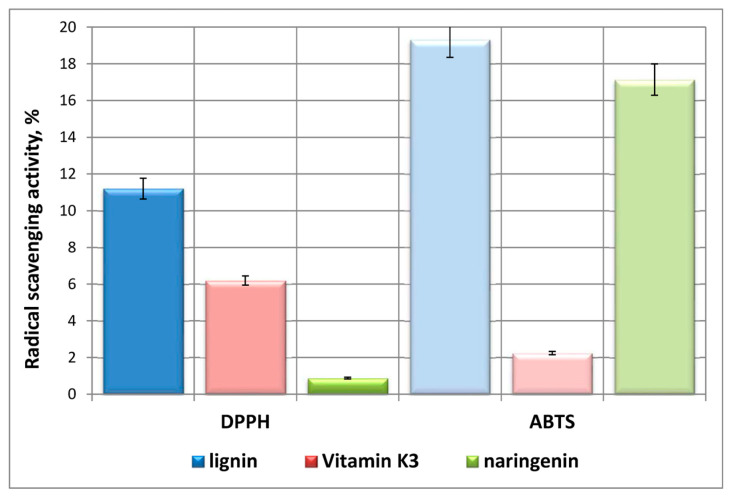
Radical scavenging activity of lignin, vitamin K_3_, and naringenin solutions with equal concentration of 100 mg/L.

The experimental results of the DPPH and ABTS radical scavenging activities of solutions of the pure substances vitamin K_3_ (in EtOH), naringenin (in EtOH), and lignin (in Milli-Q Water) with concentrations of 100 mg/L are presented in Figure 1. Obviously, the heterobiopolymer was characterized with the highest antioxidant capacity, which was proven by the results obtained from both applied analytical assays. The potential of the flavonoid naringenin towards scavenging of ABTS radicals overlapped with approximately 19.5 times the activity towards DPPH radicals, while the relationship for vitamin K_3_ was reversed—the DPPH potential surpassed with 64% the ABTS activity. The observed deviations in the antioxidant capacities of the three bioactive substances determined by both assays could be due to their molecular structural characteristics as well as the mechanism of the analytical methodology applied. 

According to the scientific literature, the DPPH and ABTS assays are influenced by various molecular structural characteristics and the radical scavenging potential of phenolic compounds depends on the number and position of aliphatic/aromatic hydroxyl groups on the presence of other substituents and functional groups [36,37]. Platzer et al. (2022) and Duan et al. (2022) stated that an increased number of ortho- and para-orientated Ar—OH and —OCH_3_ groups at the aromatic ring influence positively the DPPH and ABTS activities, as long as no steric hindrance occurred [37,38].

Tavares et al. (2022) established that non-etherified aliphatic and/or phenolic —OH groups, ortho—OCH_3_ groups, and the double C=C bond between the outermost carbon atoms in the side chain of lignin macromolecule contribute to its radical scavenging ability [39].

Concerning the mechanism of the DPPH assay, the DPPH radical reacts both with electron and hydrogen donors, i.e., the method is based on both electron transfer and hydrogen atom transfer pathways [40].

The present study observed approximately six times higher DPPH potential of vitamin K_3_ as compared to that of naringenin and a reversed relationship with regards to the ABTS radical scavenging activity of menadione and the flavonoid. Vitamin K_3_ is capable of one-electron reduction, leading to the formation of unstable free radical substances, which, as a result of rapid reaction with O_2_, can form ROS and cause oxidative stress. However, according to Talcott et al. (1985), one of the reduced hydroquinone-containing forms of vitamin K_3_ could act as an antioxidant [3,11]. In this respect, a team of scientists established that the hydroquinone was 10 times as potent as α-tocopherol in the reaction with phenoxy radicals [12], while, according to Vervoort et al. (1997), the synthetic vitamin exhibited a potential 100 times that of ubiquinol in regenerating vitamin E from its radical [13].

Moreover, the higher DPPH radical scavenging activity of vitamin K_3_ as compared to that of naringenin indicated that an increase in the number of phenolic hydroxyl groups did not necessarily lead to higher antioxidant potential values. Similar results were observed by Ivanova et al. (2023) [32].

Naringenin molecule is comprised of two –OH groups at positions 5 and 7 of the A aromatic ring, a hydroxyl group at 4′ position of the B aromatic ring, and a carbonyl group at position 4 of the C ring. These structural characteristics explain the ability of the flavonoid to quench free radicals and ROS and determine the natural molecule as a potent antioxidant with health-promoting properties [31]. However, according to the study of Shubina et al. (2021), the absence of –OH group at the 3′ position of the B ring in naringenin molecule provokes a significant reduction in the flavonoid antiradical activity [41].

The experimental data obtained in the present study, together with the diversions in the cited results, could be elucidated by the fact that naringenin polyphenol structure determines its sensitivity to changes in the medium. These alterations could influence the hydrophobicity, planarity of the molecule, and electrostatic interactions, which, in turn, could eventually result in changes in its antioxidant properties [22].

The antioxidant activity of a combination of biologically active compounds depends on several factors, including chemical structure, ratios and concentrations of the components, as well as the specific mechanisms involved. It is worth noting that lignin itself possesses antioxidant properties due to its polyphenolic structure, which allows it to scavenge free radicals. When it comes to its double or triple combinations with vitamin K_3_ or/and naringenin, it is plausible that they could exert synergistic antioxidant effects. By combining their respective antioxidant mechanisms, they may enhance the overall antioxidant capacity. The ability of naringenin to scavenge free radicals and the potential of vitamin K_3_ to support antioxidant enzyme activity could complement each other.

**Figure 2:**
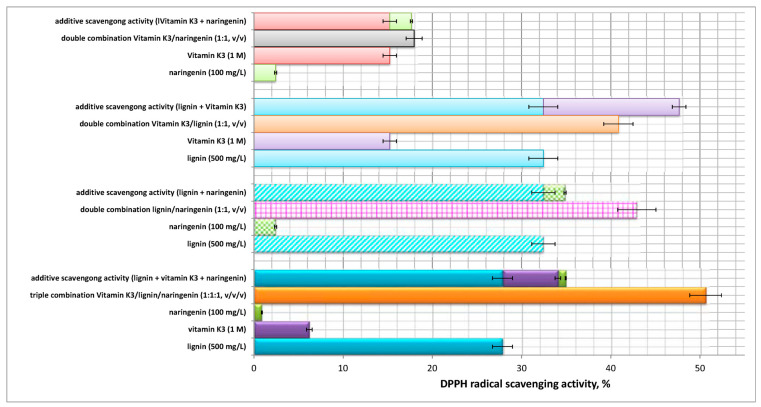
DPPH radical scavenging activity of the pure substances vitamin K_3_, lignin, and naringenin, of the binary systems vitamin K_3_/lignin and vitamin K_3_/naringenin, and of the three-component system vitamin K_3_/flavonoid/lignin.

**Figure 3:**
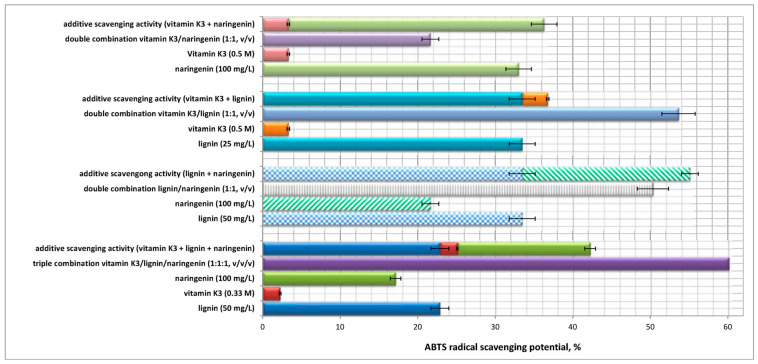
ABTS radical scavenging activity of the pure substances vitamin K_3_, lignin, and naringenin, of the binary systems vitamin K_3_/lignin and vitamin K_3_/naringenin, and of the three-component system vitamin K_3_/flavonoid/lignin.

Studies on the DPPH and ABTS radical scavenging potential of the two-component systems lignin/vitamin K_3_ and vitamin K_3_/naringenin and of the triple combination vitamin K_3_/flavonoid/lignin were carried out and the experimental data obtained are presented graphically in Figure 2 and Figure 3. The statistical significance (*p*-values matrix) of the experimental data for the DPPH and ABTS radical scavenging activity of the single, two- and three-component solutions are presented in Tables S1 and S2.

Although we studied the antioxidant activity of the single-component solutions of vitamin K_3_, naringenin, and lignin, the mechanisms of their mutual action, when combined in two-/three-component systems, remain unclear and/or undefined. An important factor that should be considered is their mutual interaction, which can be synergistic, antagonistic, or additive (no interaction). Thus, the interactions between the single components in the double and triple combinations were determined by calculation of the difference (%) by Equation (1). A comparison between the theoretical and experimental ABTS and DPPH activity values and the interaction of equimolar biopolymer/vitamin/flavonoid mixtures (% difference) are presented in Table 1. Positive values of the difference (%) are indicative of the existence of a potential synergistic effect, while negative values define antagonism. An additive effect was considered for difference approximately equal to zero when an absence of interaction could be contemplated [42].

The initial assessments performed by the most popular DPPH batch test showed that, although the flavanone naringenin seemed a very weak antioxidant, it increased the antioxidant activity of the two-component mixture with lignin and of the triple combination with the heteropolymer and vitamin K_3_ in a synergistic manner. Similar results were reported by Baranowska et al. (2021), who established that, despite the negligible reactivity towards DPPH, the flavanone naringenin, which, by itself, exhibited no redox properties within the reaction period, significantly increased the total antioxidant activity of its mixtures with quercetin and rutin [34]. An additive effect was observed for the scavenging potential of the combination vitamin K_3_/naringenin, while the pro-vitamin influenced negatively the antioxidant activity of the heterobiopolymer lignin. The latter statement was proven by the observed antagonism with respect to the overall capacity of the double combination (Figure 2, Table 1).

The ABTS radical scavenging potential of the double combination lignin/naringenin was significantly improved by the addition of the synthetic vitamin K_3_ (Figure 3). The high difference value (Table 1), which is indicative of strong interaction between the three components, as well as of synergistic effect on the overall antioxidant capacity of the three-component mixture, served as a proof of the concept. A similar tendency was observed for the double combination lignin/vitamin K_3_—as the overall ABTS scavenging potential surpassed with 31.5% the theoretical additive capacity of the two-component mixture.

Deviations were established with regards to the antioxidant activity of the combinations: vitamin K_3_/lignin and naringenin/lignin determined by both assays. All these observations suggest that, when considering redox-related biological activities of individual bioactive compounds versus their mixtures, the interactions between components must be taken into account. Based on the present experimental results, as well as on other modern scientific investigations, the growing complexity of mixtures of phytochemicals and vitamins appear to create novel redox active compounds rather than “boost” the mixture with new bioactivities, characteristic of the components added, which could be inferred by the concept of synergy [34].

The antioxidant activity of phenolic compounds is dependent on the pH of the medium, since changes in pK_a_ values are ascribed to alterations in ionization hydroxyl groups or other functional groups. According to Ghosh et al. (2015), the antioxidant potential is influenced by pH due to (i) electrochemical oxidation; (ii) involvement of H^+^; (iii) the stability of the compound to oxidation processes; (iv) compound transformation; and (v) oxidation rate [43]. Thus, the effect of pH on the antioxidant properties of the studied single-, double-, and triple-component solutions has to be determined and analyzed.

**Figure 4:**
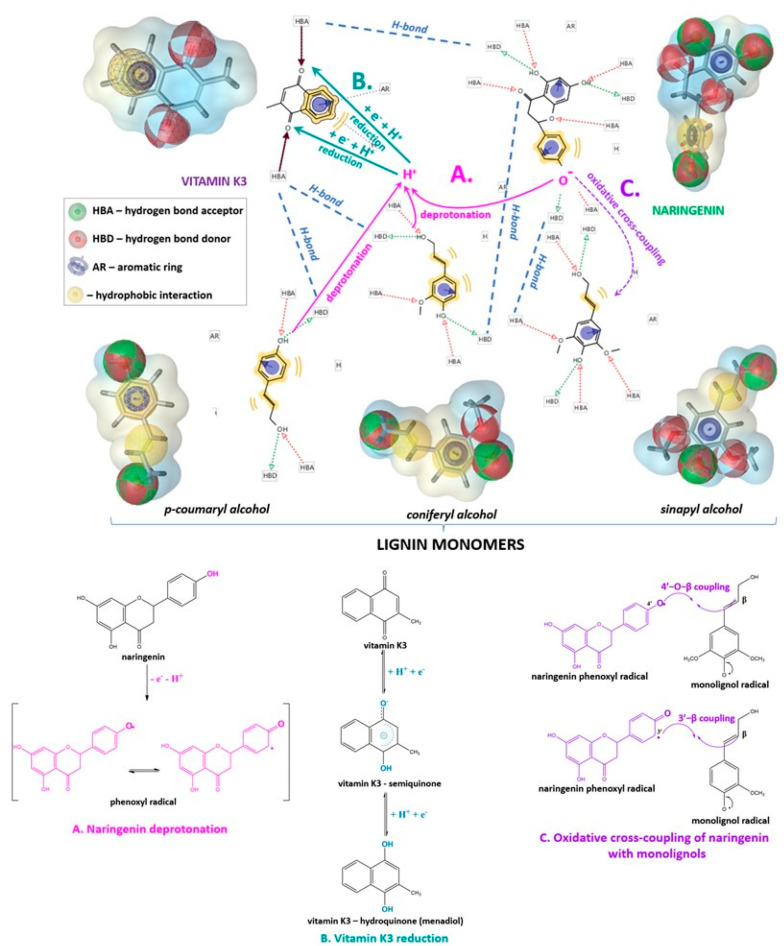
Schematic representation of the probable intermolecular interactions, deprotonation, reduction, and oxidative cross-coupling reactions between naringenin, vitamin K_3_, and lignin in their two- and three-component combinations [50,51].

Lignin is a complex natural polycyclic polymer that is formed from three alcohol monomers called monolignols, namelycoumaryl, coniferyl, and sinapyl alcohols. The heterobiopolymer macromolecules contain various functional groups and many negatively charged substituents, which determine its capability of participation in intermolecular interactions, as well as its high reactivity. The alkali lignin applied in the present investigations was dissolved in Milli-Q water and the pH of its solutions was alkaline (Table 2). According to our previous study, an increase in pH leads to an increase in the net negative charge of lignin due to intensified deprotonation of phenolic –OH groups, which, in turn, results in increased concentrations of H^+^ (Figure 4), reducing the pH on the surface of the heteropolymer [44].

The bioactivity of flavonoids depends on various parameters, such as hydroxyl groups acidity, proton affinity of phenoxide anions, ionization potential, and phenolic O–H bond dissociation [45,46,47]. With respect to the proposed mechanisms, which explain the free radical scavenging ability of antioxidants, obviously the dissociation of the phenolic O–H bonds is responsible for the formation of the less reactive flavonoid radicals in the hydrogen atom transfer mechanism. A deprotonation of the bioactive molecule followed by rapid electron transfer to scavenge ROS is involved in the sequential proton loss electron transfer. The single electron transfer followed by proton transfer mechanism starts with the formation of phenoxy radical cations by electron abstraction from the neutral molecule of the flavonoid. Therefore, the values of the dissociation constants of the hydroxyl groups and the pH of the medium are important parameters that affect the antioxidative capacity of flavonoids. According to the study of Farajtabar and Gharib (2012), the pK_a_ constants of naringenin are pK_a1_ = 7.13 for the 7-OH group, pK_a2_ = 8.63 of the 5–OH group, and pK_a3_ = 9.82 of the 4′–OH group [24]. Consequently, in the double or triple combinations where pH > pK_ai_ (i = 1, 2, 3), the respective –OH groups of naringenin are deprotonated, forming active radicals (Figure 4).

The role of vitamin K_3_ in promoting the overall DPPH and ABTS scavenging potential of the triple combination was probably due to its participation in a two-stage redox process with the deprotonated by naringenin and lignin macromolecules H^+^ comprised of the formation of a semiquinone in a quasi-reversible first stage, followed by a second reaction, associated with a radical dianion formation (Figure 4). Moreover, the hydroquinone possesses stronger basic character and is featured with higher reactivity as compared to the semiquinone due to its liability to undergo a deprotonation process or to participate in acid–base reactions [48,49].

Scientific studies report the participation of flavonoids in oxidative radical cross-coupling reactions with monolignols, which leads to the formation of flavono-lignan moieties comprised of phenylpropanoid units bound through various types of linkages [34,50,51,52]. Similar interactions via free radical coupling mechanisms and the formation predominantly of 4′–O–β and 3′–β coupling linkages are proposed in the present study between naringenin molecules and lignin monomers in the two- and three-component mixtures (Figure 4).

Based on the results of the present study, which are in conformity with the observations of other authors, it could be confirmed that the antioxidant potential of the complex flavonoid/vitamin/biopolymer conjugated systems and the impact (additive, antagonistic, and synergistic) of the individual components on the overall radical scavenging activity depends on the physicochemical properties and molecular structural characteristics, such as number, arrangement, and mutual position of –OH, –OCH_3_, and =C=O groups. However, other properties like dissociation, resonance, ionization, solvent type, solvation effects, intermolecular interactions, intramolecular hydrogen bonds, bond dissociation, etc., have to be considered as well [42,53,54].

## 3. Materials and Methods

### 3.1. Chemicals

The following reagents: (±)-Naringenin (C_15_H_12_O_5_, CAS No.: 67604-48-2, ≥95%), lignin (alkali, CAS No.: 8068-05-1), menadione (vitamin K_3_) (C_11_H_8_O_2_, CAS No.: 58-27-5), DPPH (2,2-Diphenyl-1-(2,4,6-trinitrophenyl)hydrazyl, C_18_H_12_N_5_O_6_, CAS No.: 1898-66-4), ABTS (ABTS™ chromophore, diammonium salt, C_18_H_18_N_4_O_6_S_4_·(NH_3_)_2_, CAS No.: 30931-67-0), ethanol (EtOH, C_2_H_5_OH, p.a. ≥ 99.8%), and K_2_S_2_O_8_ (CAS No.: 7727-21-1ACS reagent, ≥99.0%), supplied by Sigma-Aldrich (St. Louis, MA, USA), were applied in the present investigations.

### 3.2. Radical Scavenging Potential

The DPPH scavenging activity of the individual compounds and their two- and three-component combinations was determined by an adapted method [55]. In brief, 3 mL of 0.1 mM DPPH ethanol solution was mixed with 200 µL of the corresponding sample and the mixture, protected from light, was stirred on DLAB MS7-H550-S magnetic hot plate stirrer (DLAB SCIENTIFIC Co., Ltd., Beijing, China) for 30 min. The absorbance was measured at λ = 517 nm. DPPH-scavenging activity (DPPH, %) was calculated according to [32].

The ABTS assay is based on the generation of ABTS•^+^ cation radical as a result of the reaction between equal volumes of ABTS aqueous solution (7 mM) and K_2_S_2_O_8_ solution (2.4 mM) for 24 h at a temperature of 20 ± 2 °C in the dark. Then, the final solution was diluted with 99.8% EtOH so that absorbance of 0.700 at λ = 734 nm was reached. The method comprised of the addition of 200 µL sample to 3.6 mL of ABTS solution and spectrophotometric measurement of the absorbance at the same wavelength. The radical scavenging activity is reported as ABTS radical inhibition (%) [56], calculated according to [32].

UV/Vis spectrophotometer DR 5000 (Hach Lange, Düsseldorf, Germany), supplied with 10 mm quartz cuvette cells, was used for the spectrophotometric measurements. pH measurements were made on Consort C931 pH-meter (Consort, Turnhout, Belgium).

### 3.3. Intermolecular Interactions and Molecular Docking

The interactions between the flavonoid, the biopolymer, and the pro-vitamin were described as the difference in the experimentally determined and theoretical (calculated) antioxidant activity values by Equation (1) [42]:

(1) $$Difference\;\left(\%\right)=\left(\frac{{AA}_{abc}}{{AA}_{a}+{AA}_{b}+{AA}_{c}}\times 100\right)-100$$

where *AA_abc_* is the experimentally obtained antioxidant activity of the double or triple combination; *AA_a_*, *AA_b_*, *AA_c_*—the experimental antioxidant activities of the individual components.

The theoretical values for each combination were calculated by dividing the experimental values by the number of compounds in the mixtures.

The obtained interactions (% difference) were used to determine the potential synergistic (positive values, difference, % > 0), antagonistic (negative values, difference, % < 0), or additive (difference, %~0, absence of interaction) effects.

The intermolecular interactions between naringenin, vitamin K_3_, and lignin in their two- and three-component combinations were predicted and modeled on the basis of a molecular docking concept performed with the pharmacophore modeling and screening software program LigandScout 4.4.8 (InteLigand GmbH, Wien, Austria).

### 3.4. Statistical Analyses

The data obtained from the DPPH and ABTS radical scavenging studies were expressed as means ± standard deviations (SD) from three repetitions. The statistical significance of the experimental results was determined by performing a Student’s *t*-test and ANOVA test as the post hoc tests accomplished using XLSTAT Version 2022.4.5. statistical software for Excel (Microsoft Corporation, Redmond, WA, USA). A value of *p* < 0.05 was considered statistically significant.

## 4. Conclusions

The flavonoid naringenin increased the DPPH antioxidant potential of its two-component mixture with lignin and of the triple combination with the heteropolymer and vitamin K_3_ in a synergistic mode, despite the weak antioxidant activity of the pure bioflavonoid. An additive effect was observed for the radical scavenging potential of the combination vitamin K_3_/naringenin, while the pro-vitamin affected negatively the activity of the heterobiopolymer. The latter was substantiated by the observed antagonism with respect to the overall capacity of the double combination.

The ABTS radical scavenging potential of the two-component system lignin/naringenin was significantly improved by the addition of vitamin K_3_, which resulted in strong interaction between the three components and led to an outlined synergistic effect on the overall antioxidant capacity of the three-component mixture.

The role of vitamin K_3_ in promoting the overall DPPH and ABTS scavenging potential of the triple combination was probably due to its participation in a two-stage redox process with the deprotonated by naringenin and lignin macromolecules H^+^ comprises of the formation of semiquinone in a quasi-reversible first stage followed by a second reaction associated with the formation of a dianion radical.

Future studies are going to be directed towards defining the practical applicability of the tested pro-vitamin/flavonoid/heterobiopolymer combinations for biomedical purposes by investigations of their in vitro antimicrobial potential and in vivo biological activities on real animal models.

## Figures and Tables

**Table 1:** Comparison of theoretical and experimental ABTS and DPPH activity values and the interaction of equimolar biopolymer/vitamin/flavonoid mixtures (% difference).

Radical Scavenging Activity	ABTS Potential			DPPH Potential		
System	Experimental	Theoretical	Difference, %	Experimental	Theoretical	Difference, %
double combinations						
lignin/naringenin	50.34	27.55	−8.64	42.92	17.43	23.12
vitamin K_3_/lignin	53.64	18.385	45.89	40.85	23.83	−14.28
vitamin K_3_/naringenin	31.36	12.46	25.80	17.95	8.82	1.75
triple combination						
lignin/naringenin/vitamin K_3_	60.11	14.08	42.31	50.63	11.65	44.90

**Table 2:** pH of naringenin, lignin, and vitamin K_3_ single-, two-, and three-component solutions.

Solution Component	Solvent	Concentration/ Volumetric Ratio	pH
naringenin	EtOH	100 mg/L	6.84
lignin	Milli-Q water	500 mg/L	9.90
vitamin K_3_	EtOH	1.00 M	7.35
naringenin/vitamin K_3_	EtOH	1:1, *v*/*v*	7.60
naringenin/lignin	EtOH/Milli-Q water	1:1, *v*/*v*	8.94
vitamin K_3_/lignin	EtOH/Milli-Q water	1:1, *v*/*v*	9.80
naringenin/vitamin K_3_/lignin	EtOH/Milli-Q water	1:1:1, *v*/*v*/*v*	9.59

## Data Availability

The data presented in this study are available on request from the corresponding author. The data are not publicly available due to privacy.

## Supplementary Materials

The supporting information can be downloaded at: https://www.mdpi.com/article/10.3390/ijms242216268/s1.

## References

[1] Popa D.-S., Bigman G., Rusu M.E. (2021). The Role of Vitamin K in Humans: Implication in Aging and Age-Associated Diseases. Antioxidants.

[2] Prasad C.V., Nayak V.L., Ramakrishna S., Mallavadhani U.V. (2018). Novel menadione hybrids: Synthesis, anticancer activity, and cell-based studies. Chem. Biol. Drug Des..

[3] Fu J., Xu W., Mai K., Zhang W., Feng X., Liufu Z. (2012). Effects of dietary menadione on the activity of antioxidant enzymes in abalone, Haliotis discus hannai Ino. Chin. J. Oceanol. Limnol..

[4] Bajor M., Graczyk-Jarzynka A., Marhelava K., Kurkowiak M., Rahman A., Aura C., Russell N., Zych A.O., Firczuk M., Winiarska M. (2020). Triple Combination of Ascorbate, Menadione and the Inhibition of Peroxiredoxin-1 Produces Synergistic Cytotoxic Effects in Triple-Negative Breast Cancer Cells. Antioxidants.

[5] Lee M.H., Yang J.Y., Cho Y., Woo H.J., Kwon H.J., Kim D.H., Park M., Moon C., Yeon M.J., Kim H.W. (2019). Inhibitory Effects of Menadione on Helicobacter pylori Growth and Helicobacter pylori-Induced Inflammation via NF-κB Inhibition. Int. J. Mol. Sci..

[6] Afianda F., Wiraswati H.L., Alisjahbana B., Fauziah N. (2020). Antimalarial Effects of Menadione on Plasmodium falciparum FCR-3 Strains: In Vitro Study. IOSR J. Pharm. Biol. Sci..

[7] Kapadia G.J., Soares I.A.O., Rao G.S., Badoco F.R., Furtado R.A., Correa M.B., Tavares D.C., Cunha W.R., Magalhães L.G. (2017). Antiparasitic activity of menadione (vitamin K3) against Schistosoma mansoni in BABL/c mice. Acta Trop..

[8] de Souza A.C., Ribeiro R.C.B., Costa D.C.S., Pauli F.P., Pinho D.R., de Moraes M.G., da Silva F.C., Forezi L.S.M., Ferreira V.F. (2022). Menadione: A platform and a target to valuable compounds synthesis. Beilstein J. Org. Chem..

[9] Combs G.F., McClung J.P., Vitamin K. (2017). The Vitamins, Fundamental Aspects in Nutrition and Health.

[10] Tampo Y., Yonaha M. (1996). Enzymatic and molecular aspects of the antioxidant effect of menadione in hepatic microsomes. Arch. Biochem. Biophys..

[11] Talcott R.E., Smith M.T., Giannini D.D. (1985). Inhibition of microsomal lipid peroxidation by naphthoquinones: Structure-activity relationships and possible mechanisms of action. Arch. Biochem. Biophys..

[12] Mukai K., Itoh S., Morimoto H. (1992). Stopped-flow kinetic study of vitamin E regeneration with biological hydroquinones (reduced forms of ubiquinone, vitamin K, and tocopherol quinone) in solution. J. Biol. Chem..

[13] Vervoort L.M., Ronden J.E., Thijssen H.H. (1997). The potent antioxidant activity of the vitamin K cycle in microsomal lipid peroxidation. Biochem. Pharmacol..

[14] Liu X., Abraham M.H., Acree W.E. (2021). Descriptors for vitamin K3 (menadione); calculation of biological and physicochemical properties. J. Mol. Liq..

[15] Li L., Liu Z., Fang B., Xu J., Dong X., Yang L., Zhang Z., Guo S., Ding B. (2022). Effects of Vitamin A and K3 on Immune Function and Intestinal Antioxidant Capacity of Aged Laying Hens. Braz. J. Poult. Sci..

[16] Booth S.L. (2009). Roles for vitamin K beyond coagulation. Annu. Rev. Nutr..

[17] Fizesan I., Rusu M.E., Georgiu C., Pop A., Stefan M.-G., Muntean D.M., Mirel S., Vostinaru O., Kiss B., Popa D.-S. (2021). Antitussive, Antioxidant, and Anti-Inflammatory Effects of a Walnut (*Juglans regia* L.) Septum Extract Rich in Bioactive Compounds. Antioxidants.

[18] He T., Hatem E., Vernis L., Lei M., Huang M.-E. (2015). PRX1 knockdown potentiates vitamin K3 toxicity in cancer cells: A potential new therapeutic perspective for an old drug. J. Exp Clin. Cancer Res..

[19] Lamson D.W., Gu Y.H., Plaza S.M., Brignall M.S., Brinton C.A., Sadlon A.E. (2010). The Vitamin C: Vitamin K3 System—Enhancers and Inhibitors of the Anticancer Effect. Altern. Med. Rev..

[20] Bonilla-Porras A.R., Jimenez-Del-Rio M., Velez-Pardo C. (2011). Vitamin K3 and vitamin C alone or in combination induced apoptosis in leukemia cells by a similar oxidative stress signalling mechanism. Cancer Cell Int..

[21] Kaźmierczak-Barańska J., Karwowski B.T. (2022). Vitamin K Contribution to DNA Damage—Advantage or Disadvantage? A Human Health Response. Nutrients.

[22] Jabbari M., Jabbari A. (2016). Antioxidant potential and DPPH radical scavenging kinetics of water-insoluble flavonoid naringenin in aqueous solution of micelles. Colloids Surf. A Physicochem. Eng. Asp..

[23] Saiqing T., Zhen R., Axue M., Dong W., Jiushe K. (2022). Effect of vitamin K on wound healing: A systematic review and meta-analysis based on preclinical studies. Front. Pharmacol..

[24] Farajtabar A., Gharib F. (2013). Spectral analysis of naringenin deprotonation in aqueous ethanol solutions. Chem. Pap..

[25] Salehi B., Fokou P.V.T., Sharifi-Rad M., Zucca P., Pezzani R., Martins N., Sharifi-Rad J. (2019). The Therapeutic Potential of Naringenin: A Review of Clinical Trials. Pharmaceuticals.

[26] Jayaraman J., Jesudoss V.A., Menon V.P., Namasivayam N. (2012). Anti-inflammatory role of naringenin in rats with ethanol induced liver injury. Toxicol. Mech. Methods.

[27] Hua F.Z., Ying J., Zhang J., Wang X.F., Hu Y.H., Liang Y.P., Liu Q., Xu G.H. (2016). Naringenin pre-treatment inhibits neuroapoptosis and ameliorates cognitive impairment in rats exposed to isoflurane anesthesia by regulating the PI3/AKT/PTEN signalling pathway and suppressing NF-kappab-mediated inflammation. Int. J. Mol. Med..

[28] Ali R., Shahid A., Ali N., Hasan S.K., Majed F., Sultana S. (2017). Amelioration of benzo[a]pyrene-induced oxidative stress and pulmonary toxicity by naringenin in Wistar rats: A plausible role of COX-2 and NF-kappab. Hum. Exp. Toxicol..

[29] Manchope M.F., Calixto-Campos C., Coelho-Silva L., Zarpelon A.C., Pinho-Ribeiro F.A., Georgetti S.R., Baracat M.M., Casagrande R., Verri W.A. (2016). Naringenin inhibits superoxide anion-induced inflammatory pain: Role of oxidative stress, cytokines, Nrf-2 and the NO-cGMP-PKG-KATP channel signaling pathway. PLoS ONE.

[30] Park S., Lim W., Bazer F.W., Song G. (2017). Naringenin induces mitochondria-mediated apoptosis and endoplasmic reticulum stress by regulating MAPK and AKT signal transduction pathways in endometriosis cells. Mol. Hum. Reprod..

[31] Rashmi R., Magesh S.B., Ramkumar K.M., Suryanarayanan S., Rao M.V.S. (2018). Antioxidant Potential of Naringenin Helps to Protect Liver Tissue from Streptozotocin-Induced Damage. Rep. Biochem. Mol. Biol..

[32] Ivanova D., Toneva M., Simeonov E., Nikolova B., Semkova S., Antov G., Yaneva Z. (2023). Newly Synthesized Lignin Microparticles as Bioinspired Oral Drug-Delivery Vehicles: Flavonoid-Carrier Potential and In Vitro Radical-Scavenging Activity. Pharmaceutics.

[33] Fedoros E.I., Orlov A.A., Zherebker A., Gubareva E.A., Maydin M.A., Konstantinov A.I., Krasnov K.A., Karapetian R.N., Izotova E.I., Pigarev S.E. (2018). Novel water-soluble lignin derivative BP-Cx-1: Identification of components and screening of potential targets in silico and in vitro. Oncotarget.

[34] Baranowska M., Koziara Z., Suliborska K., Chrzanowski W., Wormstone M., Namieśnik J., Bartoszek A. (2021). Interactions between polyphenolic antioxidants quercetin and naringenin dictate the distinctive redox-related chemical and biological behaviour of their mixtures. Sci. Rep..

[35] Baranowska M., Suliborska K., Todorovic V., Kusznierewicz B., Chrzanowski W., Sobajic S., Bartoszek A. (2020). Interactions between bioactive components determine antioxidant, cytotoxic and nutrigenomic activity of cocoa powder extract. Free. Radic. Biol. Med..

[36] Shahidi F., Janitha P., Wanasundara P. (1992). Phenolic antioxidants. Crit. Rev. Food Sci. Nutrit..

[37] Platzer M., Kiese S., Tybussek T., Herfellner T., Schneider F., Schweiggert-Weisz U., Eisner P. (2022). Radical Scavenging Mechanisms of Phenolic Compounds: A Quantitative Structure-Property Relationship (QSPR) Study. Front. Nutr. Sec. Food Chem..

[38] Duan X., Wang X., Chen J., Liu G., Liu Y. (2022). Structural properties and antioxidation activities of lignins isolated from sequential two-step formosolv fractionation. RSC Adv..

[39] Tavares D., Cavali M., Tanobe V.d.O.A., Torres L.A.Z., Rozendo A.S., Zandoná Filho A., Soccol C.R., Woiciechowski A.L. (2022). Lignin from Residual Sawdust of *Eucalyptus* spp.—Isolation, Characterization, and Evaluation of the Antioxidant Properties. Biomass.

[40] Jabbari M., Mir H., Kanaani A., Ajloo D. (2014). Kinetic solvent effects on the reaction between flavonoid naringenin and 2,2-diphenyl-1-picrylhydrazyl radical in different aqueous solutions of ethanol: An experimental and theoretical study. J. Mol. Liq..

[41] Shubina V.S., Kozina V.I., Shatalin Y.V. (2021). Comparison of Antioxidant Properties of a Conjugate of Taxifolin with Glyoxylic Acid and Selected Flavonoids. Antioxidants.

[42] Skroza D., Šimat V., Vrdoljak L., Jolić N., Skelin A., Čagalj M., Frleta R., Generalić Mekinić I. (2022). Investigation of Antioxidant Synergisms and Antagonisms among Phenolic Acids in the Model Matrices Using FRAP and ORAC Methods. Antioxidants.

[43] Ghosh S., Chakraborty R., Raychaudhuri U. (2015). Determination of pH-dependent antioxidant activity of palm (*Borassus flabellifer*) polyphenol compounds by photoluminol and DPPH methods: A comparison of redox reaction sensitivity. 3 Biotech.

[44] Yaneva Z., Beev G., Rusenova N., Ivanova D., Tzanova M., Stoeva D., Toneva M. (2022). Antimicrobial Potential of Conjugated Lignin/Morin/Chitosan Combinations as a Function of System Complexity. Antibiotics.

[45] Klein E., Lukeš V., Ilčin M. (2007). DFT/B3LYP study of tocopherols and chromans antioxidant action energetics. Chem. Phys..

[46] Musialik M., Kuzmicz R., Pawłowski T.S., Litwinienko G. (2009). Acidity of hydroxyl groups: An overlooked influence on antiradical properties of flavonoids. J. Org. Chem..

[47] Vaganek A., Rimarcik J., Lukes V., Klein E. (2012). On the energetics of homolytic and heterolytic OAH bond cleavage in flavonoids. Comput. Theor. Chem..

[48] de Oliveira A.S., Brighente I.M.C., Lund R.G., Llanes L.C., Nunes R.J., Bretanha L.C., Yunes R.A., Carvalho P.H.A., Ribeiro J.S. (2017). Antioxidant and Antifungal Activity of Naphthoquinones Dimeric Derived from Lawsone. J. Biosci. Med..

[49] Fiorentini D., Cipollone M., Galli M.C., Landi L. (1997). Antioxidant activity of reduced menadione in solvent solution and in model membranes. Free Radic. Res..

[50] Rencoret J., Rosado M.J., Kim H., Timokhin V.I., Gutiérrez A., Bausch F., Rosenau T., Potthast A., Ralph J., Del Río J.C. (2022). Flavonoids naringenin chalcone, naringenin, dihydrotricin, and tricin are lignin monomers in papyrus. Plant Physiol..

[51] Beck R., Verrax J., Dejeans N., Taper H., Calderon P.B. (2009). Menadione reduction by pharmacological doses of ascorbate induces an oxidative stress that kills breast cancer cells. Int. J. Toxicol..

[52] Sun R.C. (2010). Cereal Straw as a Resource for Sustainable Biomaterials and Biofuels Chemistry, Extractives, Lignins, Hemicelluloses and Cellulose.

[53] Biela M., Kleinová A., Klein E. (2022). Phenolic Acids and Their Carboxylate Anions: Thermodynamics of Primary Antioxidant Action. Phytochemistry.

[54] Hang D.T.N., Hoa N.T., Bich H.N., Mechler A., Vo Q.V. (2022). The Hydroperoxyl Radical Scavenging Activity of Natural Hydroxybenzoic Acids in Oil and Aqueous Environments: Insights into the Mechanism and Kinetics. Phytochemistry.

[55] Avelelas F., Horta A., Pinto L.F.V., Cotrim Marques S., Marques Nunes P., Pedrosa R., Leandro S.M. (2019). Antifungal and Antioxidant Properties of Chitosan Polymers Obtained from Nontraditional *Polybius henslowii* Sources. Mar. Drugs.

[56] Moreno-Vásquez M.J., Plascencia-Jatomea M., Sánchez-Valdes S., Tanori-Córdova J.C., Castillo-Yañez F.J., Quintero-Reyes I.E., Graciano-Verdugo A.Z. (2021). Characterization of epigallocatechin-gallate-grafted chitosan nanoparticles and evaluation of their antibacterial and antioxidant potential. Polymers.

