# Supplementary material for: Menadione Contribution to the In Vitro Radical Scavenging Potential of Phytochemicals Naringenin and Lignin
Source: Int J Mol Sci. 2023 Nov 13;24(22):16268. doi: 10.3390/ijms242216268 (PMC10671743; doi:10.3390/ijms242216268)
Supplement: Supplementary file 1 [file ijms-24-16268-s001.zip › ijms-2679210-supplementary.pdf]

Article

Menadione Contribution to the In Vitro Radical Scavenging Potential of Phytochemicals Naringenin and Lignin

Zvezdelina Yaneva [1,*], Donika Ivanova [1], Monika Toneva [1], Milena Tsanova [2], Vanya Marutsova [3] and Neli Grozeva [2]

[1] Department of Pharmacology, Animal Physiology, Biochemistry and Chemistry, Faculty of Veterinary Medicine, Trakia University, Students Campus, 6000 Stara Zagora, Bulgaria; donika.ivanova@trakia-uni.bg (D.I.); monika.toneva@trakia-uni.bg (M.T.)
[2] Department of Biological Sciences, Faculty of Agriculture, Trakia University, Students Campus, 6000 Stara Zagora, Bulgaria; milena.tsanova@trakia-uni.bg (M.T.); n.grozeva@trakia-uni.bg (N.G.)
[3] Department of Internal Diseases, Faculty of Veterinary Medicine, Trakia University, Student Campus, 6000 Stara Zagora, Bulgaria; vanya.marutsova@trakia-uni.bg
* Correspondence: zvezdelina.yaneva@trakia-uni.bg

Table S1. Statistical significance (p-values matrix) of the experimental data for the DPPH radical scavenging activity of the single, two- and three-component solutions.

Variables	*lignin*	*naringenin*	*vitamin K_3*	*double combination lignin/naringenin (1:1, v/v)*	*double combination vitamin K_3/lignin (1:1, v/v)*	*double combination vitamin K_3/naringenin (1:1, v/v)*	*triple combination vitamin K_3/lignin/naringenin (1:1:1, v/v/v)*
lignin	0	**0.015**	0.053	0.194	0.178	0.053	**0.048**
naringenin	**0.015**	0	**0.039**	0.180	0.164	**0.039**	**0.034**
vitamin K_3	0.053	**0.039**	0	0.141	0.125	**< 0.0001**	**0.005**
double combination lignin/naringenin (1:1, v/v)	0.194	0.180	0.141	0	**0.016**	0.141	0.146
double combination vitamin K_3/lignin (1:1, v/v)	0.178	0.164	0.125	**0.016**	0	0.125	0.130
double combination vitamin K_3/naringenin (1:1, v/v)	0.053	**0.039**	**< 0.0001**	0.141	0.125	0	**0.005**
triple combination vitamin K_3/lignin/naringenin (1:1:1, v/v/v)	**0.048**	**0.034**	**0.005**	0.146	0.130	**0.005**	0

Values in bold are different from 0 with a significance level $p \leq 0.05$.

Table S2. Statistical significance (p-values matrix) of the experimental data for the ABTS radical scavenging activity of the single, two- and three-component solutions.

Variables	*lignin*	*naringenin*	*vitamin K_3*	*double combination lignin/naringenin (1:1, v/v)*	*double combination vitamin K_3/lignin (1:1, v/v)*	*double combination vitamin K_3/naringenin (1:1, v/v)*	*triple combination vitamin K_3/lignin/naringenin (1:1:1, v/v/v)*
lignin	0	**0.040**	0.069	0.094	0.084	0.117	**0.033**
naringenin	**0.040**	0	0.110	0.135	0.124	0.076	0.073
vitamin K_3	0.069	0.110	0	**0.025**	**0.014**	0.186	**0.036**
double combination lignin/naringenin (1:1, v/v)	0.094	0.135	**0.025**	0	**0.011**	0.211	0.061
double combination vitamin K_3/lignin (1:1, v/v)	0.084	0.124	**0.014**	**0.011**	0	0.201	0.051
double combination vitamin K_3/naringenin (1:1, v/v)	0.117	0.076	0.186	0.211	0.201	0	0.150
triple combination vitamin K_3/lignin/naringenin (1:1:1, v/v/v)	**0.033**	0.073	**0.036**	0.061	0.051	0.150	0

Values in bold are different from 0 with a significance level $p \leq 0.05$.
